# Co-Orientation of Replication and Transcription Preserves Genome Integrity

**DOI:** 10.1371/journal.pgen.1000810

**Published:** 2010-01-15

**Authors:** Anjana Srivatsan, Ashley Tehranchi, David M. MacAlpine, Jue D. Wang

**Affiliations:** 1 Department of Molecular and Human Genetics, Baylor College of Medicine, Houston, Texas, United States of America; 2 Department of Pharmacology and Cancer Biology, Duke University Medical Center, Durham, North Carolina, United States of America; University of Arizona, United States of America

## Abstract

In many bacteria, there is a genome-wide bias towards co-orientation of replication and transcription, with essential and/or highly-expressed genes further enriched co-directionally. We previously found that reversing this bias in the bacterium *Bacillus subtilis* slows replication elongation, and we proposed that this effect contributes to the evolutionary pressure selecting the transcription-replication co-orientation bias. This selection might have been based purely on selection for speedy replication; alternatively, the slowed replication might actually represent an average of individual replication-disruption events, each of which is counter-selected independently because genome integrity is selected. To differentiate these possibilities and define the precise forces driving this aspect of genome organization, we generated new strains with inversions either over ∼1/4 of the chromosome or at ribosomal RNA (rRNA) operons. Applying mathematical analysis to genomic microarray snapshots, we found that replication rates vary dramatically within the inverted genome. Replication is moderately impeded throughout the inverted region, which results in a small but significant competitive disadvantage in minimal medium. Importantly, replication is strongly obstructed at inverted rRNA loci in rich medium. This obstruction results in disruption of DNA replication, activation of DNA damage responses, loss of genome integrity, and cell death. Our results strongly suggest that preservation of genome integrity drives the evolution of co-orientation of replication and transcription, a conserved feature of genome organization.

## Author Summary

An important feature of genome organization is that transcription and replication are selectively co-oriented. This feature helps to avoid conflicts between head-on replication and transcription. The precise consequences of the conflict and how it affects genome organization remain to be understood. We previously found that reversing the transcription bias slows replication in the *Bacillus subtilis* genome. Here we engineered new inversions to avoid changes in other aspects of genome organization. We found that the reversed transcription bias is sufficient to decrease replication speed, and it results in lowered fitness of the inversion strains and a competitive disadvantage relative to wild-type cells in minimal medium. Further, by analyzing genomic copy-number snapshots to obtain replication speed as a function of genome position, we found that inversion of the strongly-transcribed rRNA genes obstructs replication during growth in rich medium. This confers a strong growth disadvantage to cells in rich medium, turns on DNA damage responses, and leads to cell death in a subpopulation of cells, while the surviving cells are more sensitive to genotoxic agents. Our results strongly support the hypothesis that evolution has favored co-orientation of transcription with replication, mainly to avoid these effects.

## Introduction

The fundamental processes of replication and transcription take place on the same template efficiently and accurately, requiring them to be coordinated with each other to avoid potential conflicts. In cells growing rapidly, both replication and transcription of ribosomal RNA (rRNA) genes, and many other genes, are initiated more frequently, further elevating this potential conflict [1]–[4]. Due to the asymmetry of the replisome and the transcription complex, the outcome of their encounter should depend strongly on their relative directionality. RNA polymerase (RNAP) is dislodged by replication in either direction [5],[6]. On the other hand, replication is affected mostly by head-on transcription [6]–[12].

Preventing or resolving this conflict not only requires numerous protein factors [13]–[16] but may also underlie several non-random aspects of genome organization [17],[18]. First, the highly-expressed rRNA and tRNA genes are transcribed almost exclusively co-directionally with replication across numerous species [19],[20]. Chromosomes of the bacteria *Bacillus subtilis* and *Escherichia coli* are replicated by bi-directional replication forks initiated from a single origin (*oriC*), and all rRNA operons are oriented away from *oriC* [21]–[25]. In yeast, replication fork barriers at the end of ribosomal DNA operons prevent replication from entering head-on into these strongly-transcribed regions [26]. Second, other highly-transcribed genes are also significantly enriched in the leading strand of replication in bacteria, ensuring that their transcription is co-oriented with replication [27]. This feature may be conserved in certain regions of the human genome [28]. Third, longer transcription units are enriched in the leading strand [27],[29]. Fourth, essential genes are enriched to a greater extent than non-essential genes in the leading strand [19]. Finally, there is a general bias for co-directionality of replication and transcription. In *B. subtilis* and *E. coli*, this bias is 75% and 55% of all genes, respectively [22]–[24].

Despite a general theme of avoiding head-on transcription and replication, the precise evolutionary forces shaping these inter-connected aspects of genome organization are not understood. The effect of head-on replication on transcription is proposed to impact fitness negatively by interrupting the expression of highly-transcribed genes [27], or in the case of essential genes, by leading to the formation of incomplete transcripts, which subsequently results in toxic truncated polypeptides [19]. However, the effects on replication are also deleterious. In *E. coli*, replication rate is largely unaffected by co-directional transcription, but is significantly slowed when it occurs head-on to a strong transcription unit [5],[30]. In addition, reversing transcription bias over an extended segment of the *B. subtilis* genome leads to a significant (30%) decrease of replication rate, extending the time required to replicate the chromosome and potentially impeding the cell cycle [31]. Head-on orientation of replication and transcription has been shown to result in genome instability, which can be due to obstructed replication or disrupted transcription [32]–[35]. It is proposed that the transcription of essential genes is preferentially co-oriented to lower their rate of mutagenesis [30]. Finally, apart from effects on replication and transcription, the transcription bias is also proposed to promote chromosome segregation [36],[37]. Is there a single evolutionary advantage associated with the co-directional bias? Alternatively, is the orientation of each gene selected in its own right? One challenge in understanding the evolutionary bases of orientation biases is dissecting how different aspects of genome organization are important in different circumstances and how they impact cellular fitness.

Here we report that the extent of the impact of head-on transcription on replication differs between genes within the same organism *B. subtilis*. This was dissected by creating new inversions of either an extensive region of the genome, or a localized region containing strongly-transcribed rRNA genes. Using quantitative genomic approaches, we observed differential rates of replication throughout the genome of the inversion strains—normal replication in intact genomic positions, impedance of replication elongation by ∼30% within the head-on region, and strong blockage of replication at inverted rRNA operons. We further characterized the fitness cost and found that inversion of the *oriC*-proximal half of a replichore results in a small decrease in growth rate in minimal medium, but is sufficient to confer a significant competitive disadvantage. On the other hand, the replication block at rRNA operons leads to major disruption of replication, induction of the DNA damage response and cell death. We also observed that the rate of mutation of the gene *rpoB* is increased when it is transcribed head-on to replication within an extended chromosomal inversion, specifically in rich medium. Our results strongly suggest that preservation of genome integrity has contributed to evolution of the genome-wide co-directional bias and its further enrichment in highly-expressed and essential genes.

## Results

### Inversion of ∼1/4 of the *Bacillus subtilis* chromosome to reverse its transcription bias

**Figure 1:**
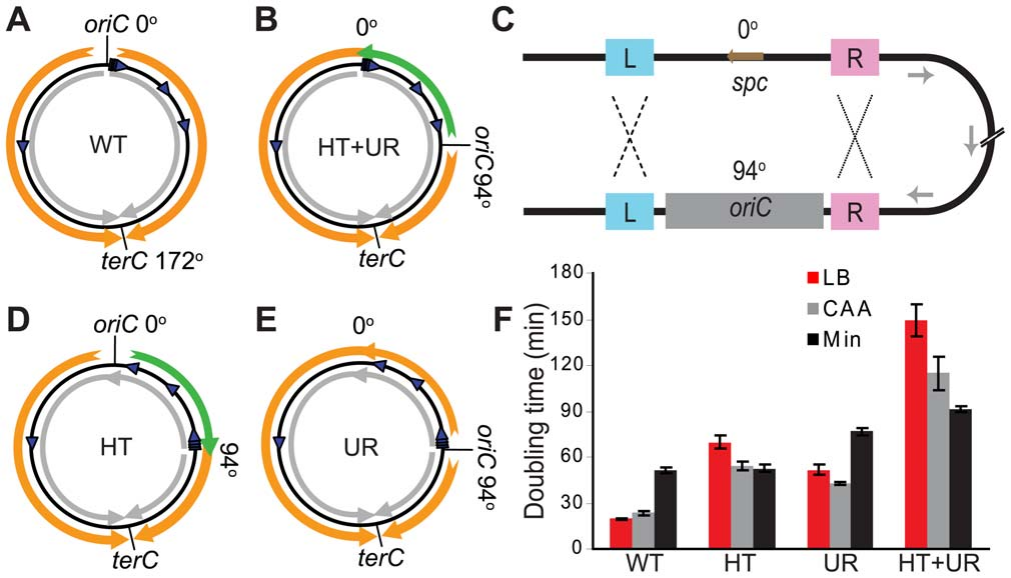
Alteration of different aspects of genome organization results in different growth defects. (A) Schematic diagram of the wild type *B. subtilis* chromosome (black circle). *oriC* at 0° and *terC* at 172° represent the origin and terminus of DNA replication, respectively. Orange arrows: replication; grey arrows: predominant direction of transcription; blue arrowheads: rRNA operons. (B) Schematic diagram of a mutant chromosome with *oriC* relocated to 94° [31], resulting in head-on transcription and unequal replichores (HT+UR). Green arrow: replication head-on to transcription between 0° and 94°. (C) Schematic diagrams of homologous recombination events leading to inversions of ¼ of the chromosome. L and R: sequences flanking *oriC* that are repeated at 0° and 94°; *spc*: spectinomycin resistance gene, which was inserted to replace the endogenous *oriC* at 0° [31]. Recombination at L (dashed lines) and R (dotted lines) give rise to the chromosomes shown schematically in (D,E), respectively. (D) Schematic diagram of the chromosome with an inversion between 0° and 94° resulting in head-on transcription (HT). (E) Schematic diagram of the chromosome with an inversion between 0° and 94° resulting in unequal replichores (UR). (F) Doubling times at 37°C in liquid LB (red bars), minimal medium with (CAA, grey bars) or without (Min, black bars) casamino acids, calculated by measuring OD_600_. Data shown are for strains in the JH642 background.

We previously moved the origin of replication (*oriC*) away from its endogenous position at 0° (Figure 1A) to 257° (not shown) or 94° (Figure 1B) to reverse the genomic transcription bias in an extended region of the chromosome, and observed that replication elongation was slowed moderately between 0° and the ectopic *oriC* position due to transcription [31]. This raised the intriguing question: what would be the potential impact of reversed transcription bias on cellular fitness and genome integrity? However, this question cannot be answered using these strains, because other aspects of their genome organization were also altered, including location of *oriC* and symmetry of the replichores (one spanning ¾ of the chromosome, the other ¼ of the chromosome). Such alterations have been shown to strongly impact cellular fitness in both *E. coli* and *B. subtilis* [38]–[40].

To examine exclusively the biological impact of reversed transcription bias, we constructed several new strains. We took advantage of the fact that the strain with repositioned *oriC* (Figure 1B) has *oriC*-flanking sequences present both at 0° and the ectopic location (Figure 1C). We reasoned that homologous recombination might occur at these repeats, and screened for such progenies (Figure 1D and 1E). Homologous recombination of repeats upstream of *oriC* (Figure 1C- repeats marked L, ∼400 bp) repositioned *oriC* to 0°, with concurrent inversion of the 0°–94° portion of the chromosome. The resulting strain had head-on transcription (HT) between 0° and 94° and equal replichore lengths (Figure 1D). Using the same strategy, we also obtained strains in which homologous recombination had taken place between repeats downstream of *oriC* (Figure 1C- repeats marked R, ∼300 bp). The resulting chromosomes have *oriC* positioned at 94° and unequal replichores (UR) but without extended regions of head-on transcription (Figure 1E). Finally, to minimize the possibility of reversion of the inversion, we removed a portion of the remnant homology region at 94°.

Using the HT strain, we evaluated the impact of head-on transcription on fitness by first examining its exponential growth (Figure 1F). The HT strain grew slowly in rich medium (LB) with a doubling time of 70 minutes, compared to 20 minutes for the control. In minimal medium however, the doubling time of the HT strain was similar to that of the control (53 and 52 minutes, respectively). In contrast, the UR strain was sicker than the control in both LB and minimal media (doubling times of 52 and 77 minutes, respectively), indicating a general growth defect due to differing replichore lengths and/or ectopic positioning of *oriC*. Therefore, unlike the general growth defect introduced by uneven replichores, the growth defect caused by inverting transcription bias over ¼ of the chromosome is nutrient-dependent.

### Head-on transcription decreases replication fork speed on a genomic scale

**Figure 2:**
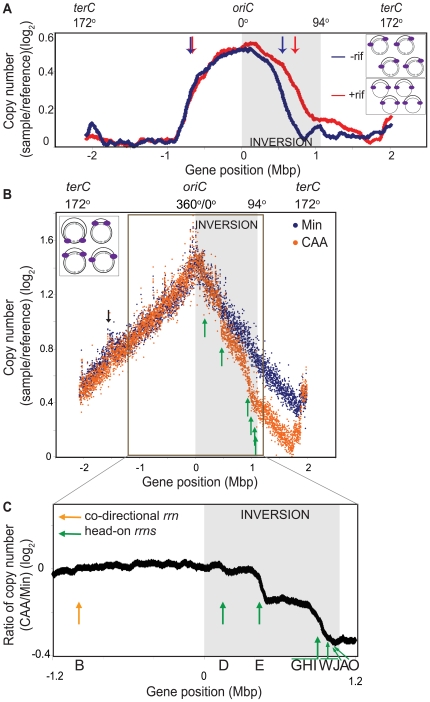
Genomic microarray profiles of the HT inversion strain. (A) Synchronized replication profile of the HT strain. Cells containing the *dnaB134*ts allele were grown in Min at 30°C and their replication cycle was synchronized by shifting to 45°C for 60 minutes, and then back to 30°C to allow replication initiation. Genomic profiles were obtained 30 minutes after initiation, relative to pre-initiation reference DNA. Profiles were obtained without (blue) or with (red) addition of rifampicin (rif) 4 minutes after initiation to inhibit transcription. Elevation of the baseline of the gene dosage profile on the right replichore is likely due to incomplete replication of the pre-initiation reference DNA of the HT strain, despite the 60 minutes incubation at 45°C. Grey shaded region: inversion; blue and red arrows: average positions of replication forks in the absence (blue) or presence (red) of rif. Insets: schematic diagram of chromosomes of the HT strain undergoing synchronized replication with (top), and without (bottom) transcription (grey arrows). Purple ovals: replisomes. (B) Overlay of the asynchronous genomic profiles of the HT strain grown in Min (blue), and CAA (orange). Profiles were obtained from asynchronous cultures grown at 37°C to OD_600_∼0.5. Average gene dosage ratios (log_2_) are plotted relative to gene positions adjusted according to deletion of the phage SPβ and the integrative and conjugative element ICE*Bs*1 clusters. The prophage-like skin element is not removed in this strain, which likely explains the small peak at −1.5 Mbp (black arrow). Green arrows: sharp changes in slope at rRNA loci in the CAA profile. Inset: schematic diagram of chromosomes of the HT strain undergoing asynchronous replication. (C) Expanded view of the ratio of gene dosage (log_2_) in CAA versus Min within the region outlined in B (−1.2 Mbp to 1.2 Mbp). Green arrows: positions of inverted rRNA operons; orange arrow: position of co-directional *rrnB*; grey shaded region: inversion.

We next examined whether head-on transcription has an effect on replication in the HT strain. We monitored synchronized fork progression in this strain using genomic microarrays (Figure 2A), in minimal medium where no significant loss of growth rate was observed (Figure 1F). Cells were synchronized for their replication cycles using a temperature-sensitive allele of the replication protein DnaB [41],[42]. The gene dosage profile obtained 30 minutes after replication initiation indicates that ∼50% of cells initiated replication. The average position of replication forks can be estimated as the midpoint of the transition between replicated and unreplicated genomic positions [43]. This position is ∼0.68 Mbp from *oriC* on the left replichore and ∼0.56 Mbp on the inverted right replichore (Figure 2A, blue arrows), indicating that replication forks move slower within the inverted region. We inhibited transcription initiation by adding the drug rifampicin 4 minutes after synchronized replication began, and found that in rifampicin-treated cells replication forks progressed further in the inverted region (∼0.72 Mbp) (Figure 2A, red arrows) compared to untreated cells (∼0.56 Mbp), demonstrating that the reduction in replication fork speed is due to transcription. This reduction in fork movement does not lead to proportionally slower growth likely because *B. subtilis* has flexible cell division cycles and can compensate for slower fork progression via multifork replication [4].

### Nutrient-dependent changes in the asynchronous gene dosage profile

We next examined whether the decreased replication rate in the inverted region varied depending on nutrient status and genomic position. To this end, we obtained the genomic microarray profile of the HT strain during exponential growth (Figure 2B). Cells are not synchronized and hence the positions of the replication forks would vary from cell to cell (Figure 2B inset). Importantly, by assuming that these cells are in a steady state and their genomic profile is time-invariant on a population basis, we can use this profile to calculate replication speed at every position on the chromosome. This speed is inversely correlated with the local slope of log values of the gene dosage with respect to gene positions (see Materials and Methods).

We first observed that in minimal medium, the genomic profile was smooth but asymmetric (Figure 2B, blue). The rates of replication were similar in the unaltered regions of the chromosome, indicated by similar slopes on the left replichore (172° to 360°, 0.408±0.002/Mbp) and the non-inverted region on the right replichore (94° to 153°, 0.462±0.007/Mbp) (Table 1). In contrast, within the inverted segment on the right replichore (0° to 94°), the slope was 0.646±0.004/Mbp, indicating a ∼30% decrease in fork speed within the head-on region, in agreement with our previous results using an ectopic *oriC* [31].

**Table 1:** Slopes of asynchronous gene dosage profiles in the HT inversion strain.

Medium	Replichore	Boundaries	Transcription bias	Slope (Mbp^−1^)(±SE)
Min	Left	*oriC* (0°)-*terC* (172°)	co-directional	0.408 (±0.002)
	Right	*oriC* (0°)-*aprE* (94°)	head-on	0.646 (±0.004)
	Right	*aprE* (94°)-*pksE* (153°)	co-directional	0.462 (±0.007)
CAA	Left	*oriC* (0°)-*terC* (172°)	co-directional	0.450 (±0.002)
	Right	*oriC* (0°)-*rrnD* (13°)	head-on	0.640 (±0.101)
	Right	*rrnD* (13°)-*rrnE* (40°)	head-on	0.615 (±0.031)
	Right	*rrnE* (40°)-*rrnGHI* (80°)	head-on	0.654 (±0.015)
	Right	*rrnGHI* (80°)-*aprE* (94°)^a^	head-on	1.51 (±0.068)
	Right	*aprE* (94°)-*pksE* (153°)	co-directional	0.456 (±0.007)

a includes four rRNA operons, all transcribed head-on to replication (*rrnJW*, *rrnA* and *rrnO*).

**SE** standard error.

Interestingly, when cells were grown in a relatively rich medium (minimal medium supplemented with casamino acids, hereafter referred to as CAA), the gene dosage profile changed sharply at specific locations on the chromosome (Figure 2B, orange). These transition points could be differentiated more clearly when we derived the relative gene dosages between the profiles in CAA vs. minimal medium (Figure 2C), and clearly corresponded to the positions of the rRNA operons within the inverted segment (Figure 2B and 2C, green arrows). The steep slopes at these transitions suggest that replication progression is strongly impeded and even stalled at these locations. Other than at rRNA loci, the genomic profile in CAA was very similar to that of minimal medium including at the intact rRNA operon on the left replichore (Figure 2C, orange arrow). Our results indicate that the genome-wide impedance of replication by reversed transcription bias other than at rRNA loci is largely uniform and unaltered by nutrient conditions. Importantly, we identify rRNA loci as positions of strong, nutrient-dependent obstruction of fork movement when they are transcribed head-on.

### Head-on transcription of rRNA disrupts replication and induces the DNA damage response

**Figure 3:**
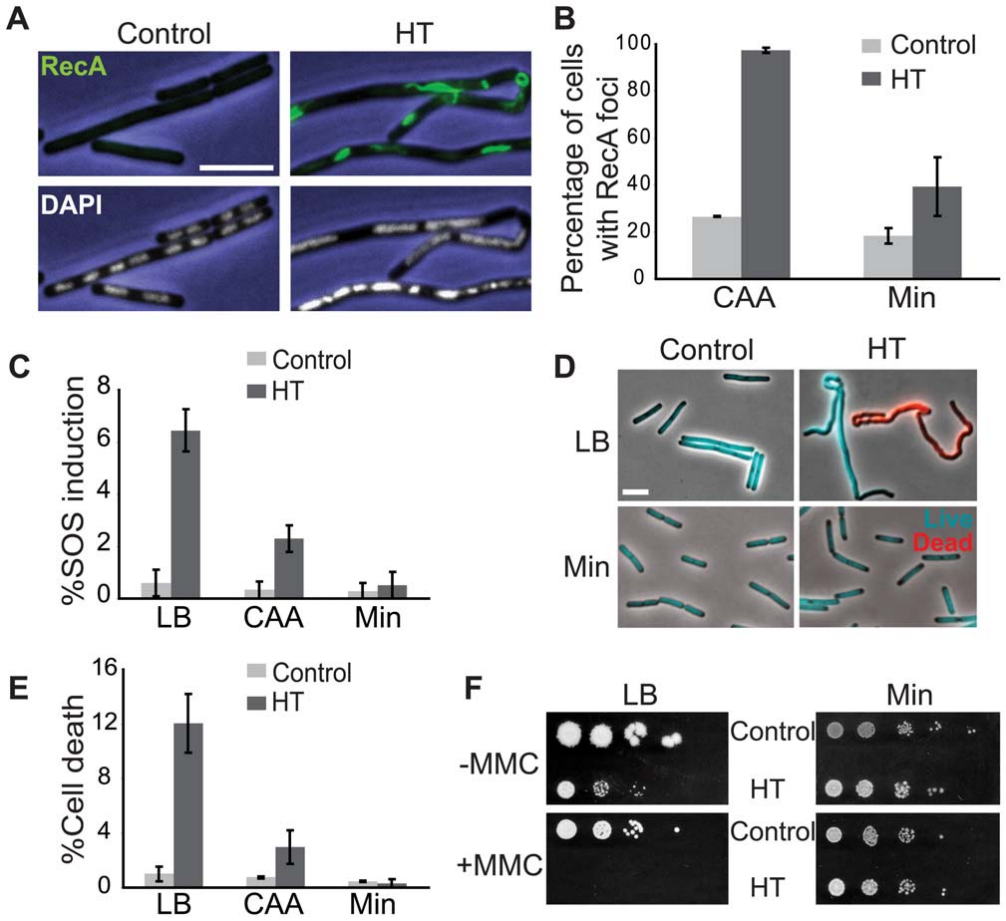
The HT strain exhibits disruption of DNA replication and loss of genome integrity. (A) RecA localization and nucleoid morphology in the HT and isogenic control strains in CAA. Upper and lower panels: phase contrast images (blue) overlaid with RecA-GFP (green) or DAPI (white) fluorescence images, respectively. (B) Percentages of cells with RecA-GFP foci in the indicated media. Cells were grown in CAA or Min and stained with DAPI and the membrane dye FM4-64 (not shown) to count individual cells. Light grey bars: control; dark grey bars: HT strain. The HT strain with *recA-gfp* was extremely sick in LB. (C) Induction of the SOS DNA damage response in the HT and isogenic control strains in the indicated media, as monitored using a TagC-GFP reporter. The total length of SOS-positive cells was divided by the total length of cells in each field. Light grey bars: control; dark grey bars: HT strain. (D) Live/dead staining of the HT and control strains in LB and Min. The fluorescent dyes SYTO9 (cyan) and propidium iodide (red) were added to exponential phase cultures (OD_600_∼0.2–0.6) to label live and dead cells, respectively. (E) Average percentages of dead cells in the control (light grey bars) and HT (dark grey bars) strains in different growth media (LB, CAA and Min). The cell length that was stained with propidium iodide was divided by the total length of cells in each field. (F) Mitomycin C (MMC) sensitivity of the HT and isogenic control strains in LB and Min. Cells were grown to OD_600_ = 0.3 and serial dilutions ranging from 10^−2^ to 10^−6^ were spotted on LB and Min plates with or without MMC (0.0625 µg/ml). Plates were scanned after overnight incubation at 37°C. Scale bar in A and D: 5 µm.

We next examined whether the forks obstructed by head-on transcription during growth in CAA were also disrupted. The recombination protein RecA localizes to stalled replication forks as foci only when the forks are disrupted [44]. Hence, we examined the sub-cellular localization of RecA using a *recA-gfp* fusion construct [45]. We visualized microscopically cells carrying this allele grown in CAA, and found that 97% of the cells of the HT strain had RecA foci compared to 27% in the control (Figure 3A and 3B), indicating that replication forks are disrupted by head-on transcription.

We also noticed that the HT strain had abnormal nucleoid morphology, which appeared filamented and even fragmented in some cases (Figure 3A, lower panels). Cell lengths also significantly increased (not shown) and chain lengths doubled (Table 2), which might explain the ∼2-fold decrease in the number of colony-forming units in strain HT compared to its control (Table 3).

**Table 2:** Increased length of chains of cells in rich medium.

Strain	Relevant genotype	Average chain length (±SE) (µm)
JDW712	Isogenic control for JDW713	23.09 (±9.38)
JDW713	Stabilized HT strain	59.19 (±6.61)
JDW858	Pre-inversion control for JDW860	15.20 (±7.56)
JDW860	*rrnIHG* inversion	26.99 (±2.44)

**SE** standard error.

**Table 3:** Colony-formation by inversion strains in different media.

Strain Relevant genotype Number of cfu/ml^a^ (±SD) (×10^8^)
		LB	Min
JDW712	Isogenic control for JDW577	0.76 (±0.3)	3.20 (±0.9)
JDW713	Stabilized HT strain	0.44 (±0.15)	5.14 (±1.6)
		**CAA** b	**Min**
JDW858	Pre-inversion control for JDW860	1.9 (±0.14)	3.6 (±0.283)
JDW860	*rrnIHG* inversion	0.293 (±0.186)	2.63 (±1.484)

a normalized with respect to OD_600_.

b JDW860 cannot grow in LB.

**cfu** colony-forming units.

**SD** standard deviation.

In addition to RecA foci formation, a subpopulation of cells exhibits the SOS DNA damage response (Figure 3C). Using a GFP-fusion reporter of *tagC*, a member of the SOS regulon [46], we found that the SOS response is induced in greater than 6% of single cells of the HT strain in LB medium, but in less than 1% in minimal medium (Figure 3C). The increase in SOS response in rich medium is accompanied by increased cell death. We performed live/dead staining in which the nucleoids of dead cells with permeable membranes stain with propidium iodide (red), while live cells stain with SYTO9 (cyan) (Figure 3D). There was a marked increase in the fraction of dead cells in the HT strain relative to the isogenic control, again specifically in rich medium (12% versus 1% of cells in LB) (Figure 3E). This suggests that failure to repair replication forks disrupted by strong head-on transcription might lead to failure to complete replication and cell death. In agreement with this hypothesis, we found that HT cells show higher sensitivity to the genotoxic agent mitomycin C in rich medium (Figure 3F), suggesting that exogenous DNA damage adds further demand on their already overwhelmed DNA repair capacity, and dramatically elevates cell death in the population.

### Increased mutation rate of a gene transcribed head-on to replication

**Figure 4:**
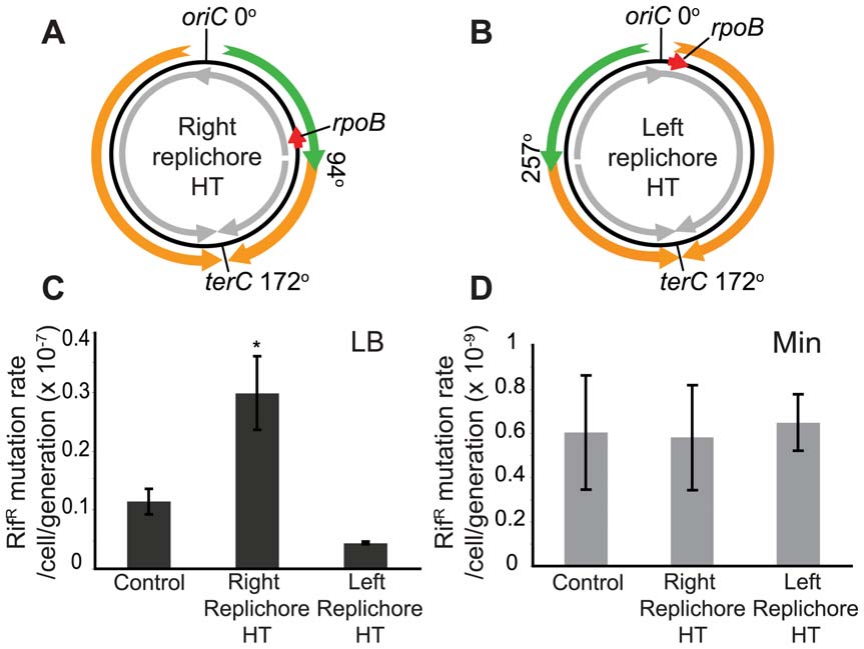
Increased mutation rate of a gene within the inversion in the HT strain. (A,B) Schematic diagrams of chromosomes (black circles) with inversion of ½ of either the right (A) or the left (B) replichore, resulting in head-on transcription (HT) within the inversion. Orange and green arrows: replication co-directional and head-on to transcription, respectively; grey arrows: predominant direction of transcription. Mutations conferring resistance to rifampicin (rif^R^) map to the *rpoB* gene (red arrow) which is transcribed co-directional to replication in the wild type and left replichore HT strains, but transcribed head-on in the right replichore HT strain. (C) Spontaneous rif^R^ mutation rates as measured by fluctuation tests in LB (dark grey bars) in the control, right and left replichore HT strains. *: rif^R^ rate in the right replichore inversion strain is significantly different from control (P<0.05). (D) Rif^R^ mutation rates in Min (light grey bars).

Having obtained evidence of disruption of replication by head-on transcription, we next examined whether it also has a consequence on genome stability by measuring the rates of mutations conferring resistance to the drug rifampicin (rif^R^). In *B. subtilis*, rif^R^ mutations map to *rpoB* [47],[48], which is transcribed co-directionally to replication in the wild-type strain but head-on in the HT strain (Figure 4A). Since *rpoB* encodes a sub-unit of RNA polymerase, its mutation might confer a growth advantage in this scenario. Hence we analyzed the results of the fluctuation test with the P_0_ method, which measures the rate of mutation independently of its effect on growth rate [49]. We observed that the mutation rate increased ∼3-fold in rich medium in the HT strain compared to an isogenic control with no inversion (Figure 4C). This could be due to a global cellular response to disruption of replication caused by the chromosomal inversion. To examine this possibility, we also monitored the rif^R^ mutation rate in a strain with inversion of ½ of the left replichore, leaving the *rpoB* region unaltered (Figure 4B). This strain has the same extent of reversed transcription bias as the HT strain, exhibits a similar nutrient-dependent growth defect (Table 4, Figure S1A), and has one inverted rRNA operon located near *oriC* after chromosome inversion, which causes replication blockage in rich medium, as monitored by microarrays (Figure S1B and S1C). However, there was no increase in the rif^R^ mutation rate in this strain (Figure 4C). Therefore the presence of an inversion alone is not sufficient to cause an increase in rif^R^ mutation rate, rather it is specific to the strain in which *rpoB* is within the inverted region. In minimal medium all strains had similar rif^R^ mutation rates (Figure 4D).

**Table 4:** Doubling times at 37°C.

Strain	Relevant genotype	Doubling times (minutes) (±SD)		
		LB	CAA	Min
JH642	Wild type control	20 (±0.8)	24 (±2.1)	52 (±3.1)
JDW423^a^	Inversion 0° to 94°, with head-on transcription	70 (±8.8)	54 (±4.2)	53 (±4)
JDW424^a^	Inversion 0° to 94°, with unequal replichores	52 (±6.2)	43 (±1.4)	77 (±4)
JDW425^a^	*oriC* at 94°: head-on transcription and unequal replichores	149 (±21)	115 (±15.6)	92 (±3.4)
JDW545^a^	Isogenic control for JDW577	25 (±1.8)	28 (±0.5)	47 (±0.5)
JDW577^a^	Stabilized inversion 0° to 94°, with head-on transcription	86 (±3)	50 (±3)	53 (±1.7)
JDW605^a^	Inversion 0° to 257°, with head-on transcription	62 (±3.6)	39 (±2.7)	58 (±5.7)
JDW712^b^	Isogenic control for JDW713	21 (±1.4)	28 (±0.4)	44 (±1.8)
JDW713^b^	Stabilized inversion 0° to 94°, with head-on transcription	72 (±4.7)	55 (±6.9)	44 (±0.6)
JDW858^b^	Pre-inversion control for *rrnIHG* inversion (*ybaN* to *rrnG-5S*)	20 (±0)	28 (±0.7)	42 (±1)
JDW859^b^	Pre-inversion control for *rrnIHG* inversion (*ybaJ* to *rrnG-5S*)	20 (±0)	28 (±0.7)	42 (±1)
JDW860^b^	*rrnIHG* inversion (*ybaN* to *rrnG*)	>160	44 (±4.9)	44 (±1.0)
JDW861^b^	*rrnIHG* inversion (*ybaJ* to *rrnG*)	>160	54 (±5.0)	46 (±4.6)

a JH642 background.

b YB886 (prophage “cured”) background.

**SD** standard deviation.

### Inversion of ribosomal RNA operons is sufficient to disrupt replication

**Figure 5:**
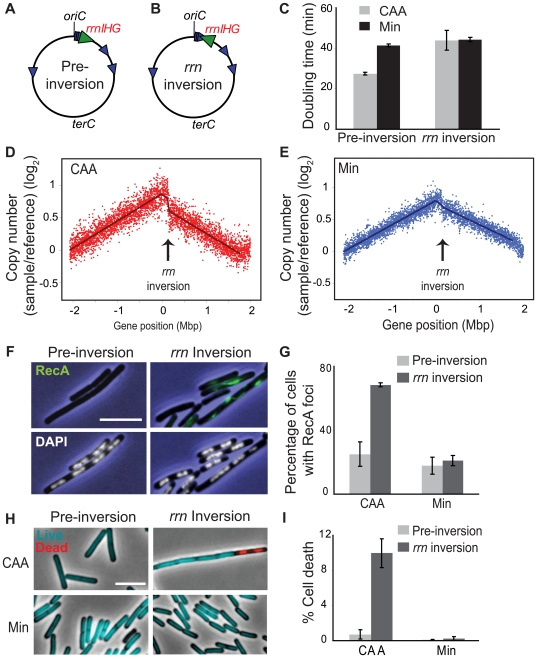
Inversion of *rrnIHG* impedes replication, triggers RecA recruitment, and elevates cell death. (A,B) Schematic diagrams of the chromosomes (black circles) of the pre-inversion control strain (A) and the *rrnIHG* (*rrn*) inversion strain (B). Blue arrowheads: rRNA operons; large green arrowhead: *rrnIHG* cluster. (C) Doubling times of the pre-inversion and *rrn* inversion strains at 37°C in CAA (grey bars) and Min (black bars). (D,E) Asynchronous gene dosage profiles of the *rrn* inversion strain in CAA (D) and Min (E). Profiles were obtained from asynchronous cultures grown at 37°C in the indicated media to OD_600_∼0.5. Average ratios of copy number (log_2_) in the *rrn* inversion strain relative to fully-replicated control are plotted against the genomic position. Arrows indicate the position of the *rrnIHG* inversion. (F) RecA recruitment and nucleoid morphology in the pre-inversion and *rrn* inversion strains in CAA, obtained as described in Figure 3A. Blue: phase contrast images; green: RecA foci; white: nucleoids stained with DAPI. (G) Average percentage of cells with RecA recruitment in the indicated media. Light grey bars: pre-inversion control; dark grey bars: *rrn* inversion. (H) Live/dead staining of the pre-inversion and *rrn* inversion strains grown in CAA and Min. Images were obtained as described in Figure 3D. Cyan: live cells; red: dead cells. (I) Average percentages of dead cells in the pre-inversion (light grey bars) and *rrn* inversion (dark grey bars) strains. The cell length that was stained with propidium iodide was divided by the total length of cells in each field. Scale bar in F and H: 5 µm.

The most dramatic reduction of replication speed due to head-on transcription occurs at the rRNA operons within the inversion (Figure 2C). This suggests that the nutrient-dependent effect on replication fork progression is mostly due to inversion of the strongly-transcribed rRNA operons. We tested this hypothesis by examining the consequences of specifically inverting rRNA operons. We inverted the *rrnIHG* cluster (∼17 kbp) that contains 3 rRNA operons and 6 tRNA genes, by inserting two overlapping halves of the neomycin resistance gene (*neo*) flanking the cluster, and selecting for recombination events that created a complete *neo* gene, similar to [50] (Figure 5A and 5B). The *rrn* inversion strain was inviable in LB and had a strong growth defect in CAA compared to the pre-inversion control (doubling times of 44 and 28 minutes, respectively), while their doubling times in minimal medium were similar (44 and 42 minutes, respectively) (Figure 5C). These results indicate that the growth defect of the HT strain was mostly due to inverted rRNA genes.

We next examined asynchronous replication in the *rrn* inversion strain using genomic microarrays, and observed impedance specifically at the inverted loci, where the gene dosage profile showed sharp discontinuity (Figure 5D and 5E). This indicates that a significant number of replication forks were stalled in this short segment. This effect was much stronger in cells replicating asynchronously in CAA than in minimal medium. Further, the majority of cells with the *rrn* inversion had RecA-GFP foci/filaments when grown in CAA (Figure 5F and 5G), indicating that strong head-on transcription at this cluster is sufficient to disrupt replication forks. Notably, the nucleoid morphology of the *rrn* inversion strain was largely normal (Figure 5F, lower panels), indicating that the gross nucleoid defects of the HT strain were not due to disruption of replication by *rrn* inversion.

Finally, the *rrn* inversion lowered cell viability. The *rrn* inversion strain had a much higher fraction of dead cells relative to the pre-inversion control in CAA (10% versus 0.7%, respectively) (Figure 5H and 5I). Thus strong head-on transcription of just rRNA and tRNA operons drastically impacts cell viability through disruption of replication.

## Discussion

Genome organization has evolved to enhance fitness, as evidenced by observations that certain genome rearrangements are not tolerated, or cause growth defects [17], [38], [39], [51]–[56], while others do not and might even be prevalent [57]. One feature of genome organization is that it precludes extensive head-on transcription [5],[17],[18],[27],[31]. In this study we engineered *B. subtilis* strains with the co-directional bias of replication and transcription reversed over either an extended segment of the genome or at a localized rRNA gene cluster. We employed microarray-based copy number profiling that enabled us to visualize directly the replication status of the entire genome, to identify positions at which there are significant perturbations and to quantify the extent of such perturbations. We found that replication is affected by head-on transcription in at least two ways: one is the apparently uniform deceleration throughout the extended region of reversed transcription bias, and the second and stronger is the disruption of replication at highly-transcribed rRNA loci. Disruption of replication at rRNA genes activates DNA repair pathways and results in sensitivity to genotoxic stress and loss of viability. Together these observations support the hypothesis that head-on collisions between transcription and replication result in loss of genome integrity, and that avoidance of this consequence contributes to the evolution of co-orientation bias in genomes.

### Other aspects of genome organization

Previously we had engineered *B. subtilis* strains in which the replichores were unequally distributed, and a significant portion of the genome was replicated by forks traveling in the opposite direction to transcription. We found that DNA replication elongation was impeded within the region of reversed transcription bias and these strains had strong growth defects [31]. However it was not possible to attribute the growth defects to reversed transcription bias alone since uneven replichores themselves have been shown in *E. coli* to lead to strong growth defects and dependence on recombination and/or DNA translocation machineries for viability [38],[39]. Therefore, we have constructed several new strains that separate the alteration of replichore symmetry from that of the transcription-replication bias (Figure 1).

We found that the *B. subtilis* HT strain with head-on transcription and replication exhibits a strong growth defect only in rich medium, and the UR strain with asymmetric replichores exhibits growth defects regardless of growth medium. The effect of both aspects of genome alteration on fitness is approximately multiplicative (Figure 1F and Table S1) [58],[59]. There are small deviations but this is significant only in minimal medium (p = 0.01). Thus we were able to largely separate two aspects of genome organization–the impact of unequal replichore size, and the impact of colliding transcription and replication forks, on genome integrity and cellular fitness.

We found that the HT strain with the extended inversion also exhibits strong disruption of replication and induction of DNA damage response in rich medium. In addition, it has altered nucleoid morphology and a long and twisted cell shape especially in rich medium (Figure 3A and 3D). We further inverted only an *rrn* cluster, and demonstrated that it is sufficient to cause disruption of replication, but not the nucleoid morphology defect. The change in morphology in the HT strain can be either associated with head-on transcription at locations other than rRNA operons, or with additional effects of inverting ¼ of the chromosome. The latter could include the alteration of gene positions relative to the origin, which affects their dosage [4],[60], ectopic localization of the *parS* sites which affect chromosome organization [61]–[63], or defects in chromosome segregation which is proposed to be facilitated by transcription [36],[37].

### Relationship between genome-wide replication rate and transcription

Using synchronized microarrays, we demonstrate that inverting the transcription bias over ¼ of the chromosome decreases fork progression rate in a transcription-dependent manner (Figure 2A), confirming our previous results obtained using an engineered strain with an ectopic *oriC* [31]. However, transcription throughout the genome is not uniform. To examine whether different inverted transcription units have different impacts on replication, we obtained asynchronous microarray profiles of exponentially growing cells (Figure 2B). Using mathematical analysis to obtain the rate of replication as a function of genomic position, we confirmed a modest genome-wide impedance of replication throughout the inverted region and showed that it is mostly independent of gene position. An important exception is at rRNA and tRNA operons, evidenced by the punctuated pattern of replication stalling at these clusters. Further, this stalling is strongly potentiated by growth in rich medium.

In general, growth in rich medium results in higher initiation frequencies of both replication and rRNA transcription [1]–[4], either of which could elevate the conflict between transcription and replication, thereby accounting for the observed increase in replication stalling. However, we observed that in the right replichore HT inversion strain, replication is not initiated more frequently in rich medium than in minimal medium. In contrast to wild type cells where gene dosage at *oriC* is much higher in rich medium than in poor medium, indicating higher rate of replication initiation [31], gene dosage at *oriC* in the HT strain is similar in CAA and minimal media (Figure 2B). The genomic profiles of the HT strain in the two media are almost identical except at the inverted *rrn* loci (Figure 2C). It is possible that failure of replication elongation prevents subsequent replication initiation; alternatively, replication initiation frequency could be lower because it is coupled to growth [3],[4], which is slower for the HT strain in rich medium (Figure 1F). Regardless of the reason, it is clear that in the inversion strains, the effect on replication observed at rRNA operons in rich medium is not due to increased replication but is exclusively due to stronger rRNA transcription, which is initiated more frequently because of higher iNTP and lower (p)ppGpp levels [64].

Several models exist to explain why replication is stalled by strong head-on transcription of rRNA operons. The replisome might be capable of bypassing a single head-on RNAP, but the presence of multiple RNAPs on the long and highly-transcribed *rrn* region could make it harder for the replisome to proceed. In addition, since rRNA operons are highly structured regions, their transcription might obstruct replication forks, as proposed for other unusually structured regions [8],[65]. RNAPs might also stall upon head-on replication to form backed-up RNAP arrays. Backed-up RNAP arrays can create a barrier to replication [15]. Finally, head-on transcription might create RNA-DNA duplexes or supercoiling of DNA that poses a barrier to replication [66]–[68], and this barrier might strengthen to the extent of blocking replication when transcription is sufficiently strong.

### Impact of head-on transcription on fitness and genome integrity

Inverting the transcriptional bias of 1/4 of the *B. subtilis* chromosome slows replication rate within this region by ∼30%. The growth rate of the HT inversion strain in minimal medium is not significantly affected (Table 4). However we discovered that the HT strain indeed has a significant growth disadvantage even in minimal medium when competing with wild type cells, with its relative fitness being 0.92 (+/−0.07) (after factoring in the marker effect) (Table S2, Text S1). This selective effect enables the wild type strain to take over after multiple generations and clearly is sufficient to shape genome evolution.

The impact of inversions on replication and cellular fitness is much stronger when cells are grown in rich media. Inverted rRNA genes in both the HT and *rrn* strains result in replication blocks (Figure 2 and Figure 5) and likely lead to extensively delayed cell-cycle progression, which explains the dramatic increase of doubling time in rich medium (Figure 1F and Figure 5C). Indeed, blockage of replication elongation has been shown to prevent cell proliferation in *E. coli*, which can only be reversed upon removal of the barrier [69]. More importantly, we obtained strong evidence that the obstruction created by inverted rRNA transcription also leads to disruption of replication. First, RecA forms foci/filaments in the majority of these cells in rich medium, indicating generation of single-stranded DNA or double-stranded ends (DSEs). Second, there is a significant increase in induction of the SOS DNA damage response [70] in the inversion strains in rich medium (Figure 3C). In *B. subtilis* the SOS response is not robustly turned on by DSEs due to efficient repair by RecN, and our observation of ∼6% of cells of the HT strain showing SOS induction agrees with the reported value [71]. Third, an increased number of cell deaths occur in the inversion strains especially in rich medium, likely due to failure to repair damaged replication forks. Finally, the inversion renders cells more sensitive to the genotoxic agent mitomycin C especially in rich medium, suggesting that the DNA repair capacity in these cells is highly compromised due to overwhelming demand, leading to detrimental consequences upon challenge by external DNA damage.

It remains unclear whether replication fork collapse [72] takes place soon after forks are stalled by head-on transcription, or only when a second round of replication forks collides with a prior round of stalled replication forks, as was demonstrated previously at the replication terminator sequence in *E. coli* [73]. It is possible that both types of collisions would lead to disruption of replication, with collision of subsequent replication forks being more costly. This might explain why inverting *rrnIHG* near the origin of replication results in a particularly strong growth defect, as the second fork encounters the stalled first fork soon after it initiates from the origin of replication. Indeed the *oriC*-proximal region is the chromosomal location of highly-expressed genes, especially those involved in macromolecular synthesis [74], and thus inversions here might be particularly detrimental.

Disruption of replication has been shown to lead to higher levels of genome instability especially in the vicinity of the disruption [73],[75],[76]. We observed an increase in *rpoB* mutation rate when the genomic region encoding *rpoB* is inverted (Figure 4). This increase is only observed when cells are grown in rich medium, implicating a dependence on the level of *rpoB* transcription or disruption of replication. It is unlikely to be solely due to a global cellular response to disruption of replication such as the SOS response, since inversion of a symmetric half of the other replichore without affecting *rpoB*, did not elevate its mutation rate despite the fact that replication is clearly inhibited by inverted *rrnB* transcription in this strain (Figure 4 and Figure S1). One possibility is that disruption of replication at *rpoB* results in recruitment of an error-prone DNA polymerase via RecA. RecA has been shown to activate directly an error-prone DNA polymerase in *E. coli* [77]. Further work will be required to differentiate this possibility from other remaining possibilities, e.g., that the increased mutation rate is due to altering *rpoB* location rather than orientation, or due to disruption of replication at the neighboring rRNA loci, or that the *rpoB* mutagenesis is due to a threshold level of the SOS response that is only met upon inversion of the right, but not the left replichore.

The impairment of replication by transcription has been shown to result in transcription- associated recombination [32] or deletion [34]. Replication orientation significantly influences the spectrum of point mutations in yeast [35]. This suggests that impairment of genome replication might also contribute to transcription-associated mutagenesis [78]–[81]. Another major mechanism which might be at play is the direct activation of the error-prone translesion polymerase via its interactions with transcription factors such as NusA [82].

### Implications for the evolution of genome organization

Our observations offer strong support for the hypothesis that the effect of transcription on replication is an important driving force in the evolution of genome organization [18]. In addition, our work suggests that the precise cost might vary depending on both the gene type and the growth environment. First, inversion of the strand bias of transcription within an extended segment of the chromosome results in a small growth defect in nutrient-poor medium yet is sufficient to confer a strong competitive disadvantage. Second, the inversion of rRNA operons leads to disruption of DNA replication, which is especially costly if cells are grown in rich medium (Figure 2, Figure 3, and Figure 5). This explains why rRNA operons are all oriented co-directionally. Although the most prominent disruptive effects we observed were at inverted rRNA operons, it is possible that these effects can also be extended to other highly-expressed or long and structured genes. Third, we also observed that mutation rate is higher for the *rpoB* gene transcribed head-on, supporting a model that co-orientation of transcription and replication of essential genes might have evolved to avoid their mutagenesis [30] (Figure 4). In addition, highly-expressed non-essential genes are also intolerant of mutagenesis since it could result in reduction of their expressivity. Thus minimizing mutagenesis may also underlie the orientation bias of highly-expressed genes.

There are considerable differences in transcription orientation biases among organisms. While low G+C Firmicutes (such as *B. subtilis*) and the Mycoplasmas have strong transcription orientation biases, other bacteria do not [18]. The widely-studied bacterium *E. coli* has only 55% co-orientation bias. Interestingly, inversion of long segments of the *E. coli* replichores including rRNA operons results in no growth defect [38]. What causes such a drastic difference in the penalty of head-on transcription? There are at least three possible explanations: differences in the composition of their replication machineries [24], non-replicative helicases [16] or transcription factors (AT and JDW unpublished). These considerable differences might underlie the differential abilities of organisms to cope with conflict between transcription and replication and thus influence the evolution of their genome organization.

## Materials and Methods

### Media and growth conditions

Cells were grown in LB or defined minimal medium (50mM MOPS) [83] with 1% glucose, supplemented with 40 µg/ml tryptophan, 40 µg/ml methionine, 40 µg/ml phenylalanine, 100 µg/ml arginine, or casamino acids (CAA) (Difco) (0.5%). Cells containing inversion of rRNA operons were maintained on 5 µg/ml kanamycin (kan). Chloramphenicol (cm), erythromycin (erm), and spectinomycin (spc) were used as described at 5, 0.5 and 40 µg/ml, respectively.

### Strains and plasmids

Standard techniques were used for genetic and molecular biological manipulations [84]. Strains used are listed in Table S3. Primer sequences are listed in Table S4. Strains were constructed in the JH642 background [85], and in the YB886 phage-defective background [86] because phage excision and duplication during stressful conditions often create localized gene copy number alterations. YB886 is cured of phage SPβ, defective for phage PBSX induction [86], and also lacks the transposon-like element ICE*Bs*1 [87].

Strain JDW704 was created by introducing an ectopic *oriC* at *aprE* (94°) in YB886 and then deleting the endogenous *oriC* (0°), using genomic DNA from JDW258 [31] and MMB703 [40], respectively. Progenies of JDW704 were screened for recombination events in sequences flanking *oriC* by PCR using oJW114/oJW135, oJW115/oJW157, oJW112/oJW75 and oJW113/oJW146. The stabilized inversion strain JDW713 was obtained by transforming JDW704 with linearized plasmid pJW247. The isogenic control was generated by transforming YB886 with linearized plasmid pJW207. The left replichore inversion strain JDW605 was created by screening progenies of MMB703 [40] by PCR using primers oJW112/oJW388, and oJW113/oJW146.

Inversion of rRNA operons was performed similarly to the method described in [50] and [88]. Briefly, two halves of the neomycin resistance gene (*neo*) overlapping by 583 bp, were inserted flanking the *rrnIHG* region in strain YB886 using plasmids pJW260 and pJW261, respectively. The strain was plated on kanamycin to select for cells in which a complete *neo* gene was created by recombination between the two halves. The inversion junctions were tested by PCR using oJW450/oJW442, and oJW452/oJW436. Genomic coordinates [22] of the *rrn* inversion are 159778 to 176408 in JDW860 and 154793 to 176408 in JDW861.

Plasmid pJW247 was constructed by cloning sequences flanking the inversion junction into the vector pUC18, on either side of the *cat* gene. The sequences were amplified using oJW206/oJW360 and oJW210/oJW211, and *cat* was amplified from pGEM*cat* [89] using oJW208/oJW209. Plasmid pJW207 was constructed similarly, except that the PCR product of oJW204/oJW205 was used instead of oJW206/oJW360. Plasmid pJW260 was constructed by cloning sequences flanking the downstream *rrn* inversion junction into the vector pUC18*erm*, on either side of the ‘*neo* fragment. The sequences were amplified using oJW434/oJW435 and oJW438/439, and ‘*neo* was amplified from pBEST502 [90] using oJW436/oJW437. Plasmid pJW261 was constructed by cloning sequences flanking the upstream *rrn* inversion junction into the vector pBEST501 [90], on either side of the *neo*’ fragment. The sequences were amplified using oJW428/oJW429 and oJW432/433, and *cat* was amplified from pGEM*cat* using oJW485/oJW486.

### Microarrays and data analysis

Strains with the *dnaB134*ts allele [41],[42] were grown in minimal medium at 30°C to OD_600_ = 0.2. Cells were shifted to 45°C for 60 minutes to prevent new initiation and allow ongoing replication to complete. The temperature was rapidly shifted down to 30°C to allow synchronized initiation of replication, and the culture was split into 2 flasks. 4 minutes after the down-shift, rifampicin was added to one flask to 0.25mg/ml. Cells were collected 30 minutes after the down-shift, mixed with an equal volume of 100% ice-cold methanol and processed for microarray analysis as described [44].

Hybridization was performed according to the Agilent Oligo aCGH protocol using custom 44K oligonucleotide Agilent microarrays. Microarrays were scanned using a GenePix 4000B scanner (Axon Instruments). Cy3 and Cy5 levels were quantified using Agilent's Feature Extraction software. Relative DNA content (log_2_ ratio of Cy3 to Cy5 levels) was plotted against the gene position on the chromosome, with the origin in the center and the terminus at each end of the x-axis. For the inversion strains, the genomic positions are rearranged to reflect genome reorganization. For synchronized microarrays, the rolling average of gene dosage ratios (log_2_) for every 200 consecutive positions was calculated from the raw data, and plotted against the mid-point of these positions.

### Calculating replication rate as a function of gene-position

Genomic microarray profiles of cells grown in mid-exponential phase were obtained by hybridizing against a synchronized reference, such that the ratios were proportional to the actual gene dosage. The data for the right replichore were analyzed based on the following (for the left replichore, the equations are identical except with negative signs):

1. During exponential growth, the total number of cells increases exponentially with cell mass doubling. Define T as the mass doubling time, *t* as the time of measurement, and N(*t*) as the total number of cells at time *t*, then
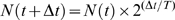
(E1)


2. Define *x* as the position of the gene, and the total gene dosage of the population of cells at position *x* as *f*(*x*, *t*). There are 4.2Mbp of nucleotides and over 10^8^ cells, so we can approximate *f* as a continuous function with continuous variables *x* and *t*, despite that fact that each cell is undergoing discrete events including replication initiation and cell division.

Assuming that cells are in steady state and their genomic profile is time-invariant on a population basis, we have
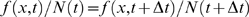
(E2)


Combining E1 and E2, we have
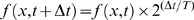
(E3)


3. The rate of replication fork progression is a function of genome position *x* but not of time *t* during steady state growth. Therefore we can define the replication rate at position *x* as *v*(*x*). By this definition, for small *Δx*, we have:
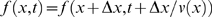
(E4)Combining E3 and E4, we have
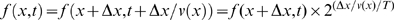
Define 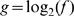, then
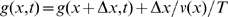
which can be rearranged as
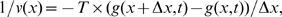
(E5)which can also be written in differential form as
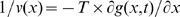
(E6)


Once we obtain *g*(*x*, *t*), equations E5 and E6 give a precise definition of how to obtain *v*(*x*). Our microarray data show that, for a fixed *t*, *g*(*x*, *t*) can be approximated by a piecewise linear function over *x*. Let a discrete series (*x_1_*, *x_2_*….*x_m_*) be the connecting points of the piecewise linear function. If *x* falls between *x_i_* and *x_i+1_*, and 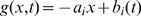, where *a_i_* is independent of *t*, then we can obtain *v*(*x*) as:
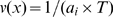
(E7)


### Measurement of mutation rates

Measurement of rifampicin-resistance (rif^R^) mutation rates was performed using the fluctuation test as described [49]. 50 parallel cultures of 1–2 ml each were set up for each strain, grown at 37°C to OD_600_∼0.5 and plated on minimal medium containing rifampicin (5 µg/ml). Serial dilutions were also plated on non-selective medium to count the number of colony-forming units. After incubation at 37°C for 36 hours, the number of plates with no rif^R^ colonies was counted. Rif^R^ mutation rate was calculated using the P_0_ method [49]. Results from 2 or more independent experiments were averaged. Error bars represent the range of data for n = 2, and the standard error for n>2.

### Live cell microscopy

The *recA::(recA-gfp spc)* allele [45] was used to replace the endogenous *recA* gene in the inversion and control strains. The *recA-gfp* strains were grown at 30°C in CAA and minimal medium. At OD_600_ = 0.2–0.6, 250 µl aliquots were labeled with the membrane dye FM4-64 (0.05 µg/ml) and/or DAPI (0.1 µg/ml). Cells were spotted onto thin agarose pads (1% agarose in 1× Spizizen's salts) on multi-well slides, covered with a cover slip and imaged in a Zeiss Axiovert microscope using a 100× oil immersion objective. Images were captured using a Hamamatsu Digital CCD camera, and analyzed using the AxioVision software. The number of cells with RecA-GFP foci or filaments relative to the total number of cells in each image was counted.

SOS induction was monitored similarly in cells containing the *tagC-gfp* reporter grown at 37°C in LB, CAA and minimal medium. Data were analyzed by calculating SOS induction per cell length.

For estimating the number of dead cells microscopically, the Live/Dead *Bac*Light Bacterial Viability Kit (Molecular Probes) was used, in which live cells are labeled with SYTO9 (green fluorescence, colored in cyan in Figure 3D and Figure 5H) and dead cells with propidium iodide (red fluorescence). Data were analyzed by calculating cell death per cell length.

For all microscopy experiments approximately 1000 cells were counted for each strain and each growth condition. Results from 2 or more independent experiments were averaged. Error bars represent the range of data for n = 2, and the standard error for n>2.

### Measurement of number of colony-forming units (cfu)

Cells were grown at 37°C to OD_600_∼0.2–0.6 and serial dilutions were plated on minimal medium. Colonies were counted after 36 hours of incubation at 37°C. Data from 3 independent experiments were averaged.

### Mitomycin C sensitivity assay

Cells were grown in either LB or minimal medium to OD_600_ = 0.3. 5 µl of 1∶10 serial dilutions ranging from 10^−2^ to 10^−6^ were spotted correspondingly on LB, Min and plates supplemented with mitomycin C to a concentration of 0.0625 µg/ml. Plates were incubated overnight at 37°C and photographed the next day. The experiment was repeated twice and representative images are shown.

## Supporting Information

Figure S1 Inversion of the *oriC*-proximal half of the left replichore (HT-left) also leads to impedance of replication fork progression. (A) HT-Left exhibits a strong growth defect especially in rich media. Doubling times at 37°C in liquid LB (red bars), minimal medium with (CAA, grey bars), or without (Min, black bars) casamino acids were calculated by measuring OD_600_. (B) Overlay of the asynchronous genomic profiles of the HT strain grown in Min (blue), and CAA (orange). Profiles were obtained from asynchronous cultures grown at 37°C to OD_600_∼0.5. Average gene dosage ratios (log_2_) are plotted relative to gene positions adjusted according to known deletions of the background strain JH642 [6]. (C) Ratios of gene dosage (log_2_) in CAA versus Min of the HT-Left strain, calculated similarly to Figure 2C. Green arrow: position of the inverted rRNA operon *rrnB*; orange arrows: positions of co-directional rRNA operons; grey shaded region: inversion.
(0.95 MB TIF)

Table S1 Comparison between the observed and expected fitness of the HT and UR mutants in the indicated growth media, under the multiplicative null model.
(0.02 MB DOC)

Table S2 Relative fitness of the HT strain in the indicated growth media (W_HT_).
(0.03 MB DOC)

Table S3 Strains.
(0.05 MB DOC)

Table S4 Primers.
(0.05 MB DOC)

Text S1 Supplemental Materials and Methods.
(0.03 MB DOC)
