# Supplementary figures and images for: Co-Orientation of Replication and Transcription Preserves Genome Integrity
Source: PLoS Genet. 2010 Jan 15;6(1):e1000810. doi: 10.1371/journal.pgen.1000810 (PMC2797598; doi:10.1371/journal.pgen.1000810)

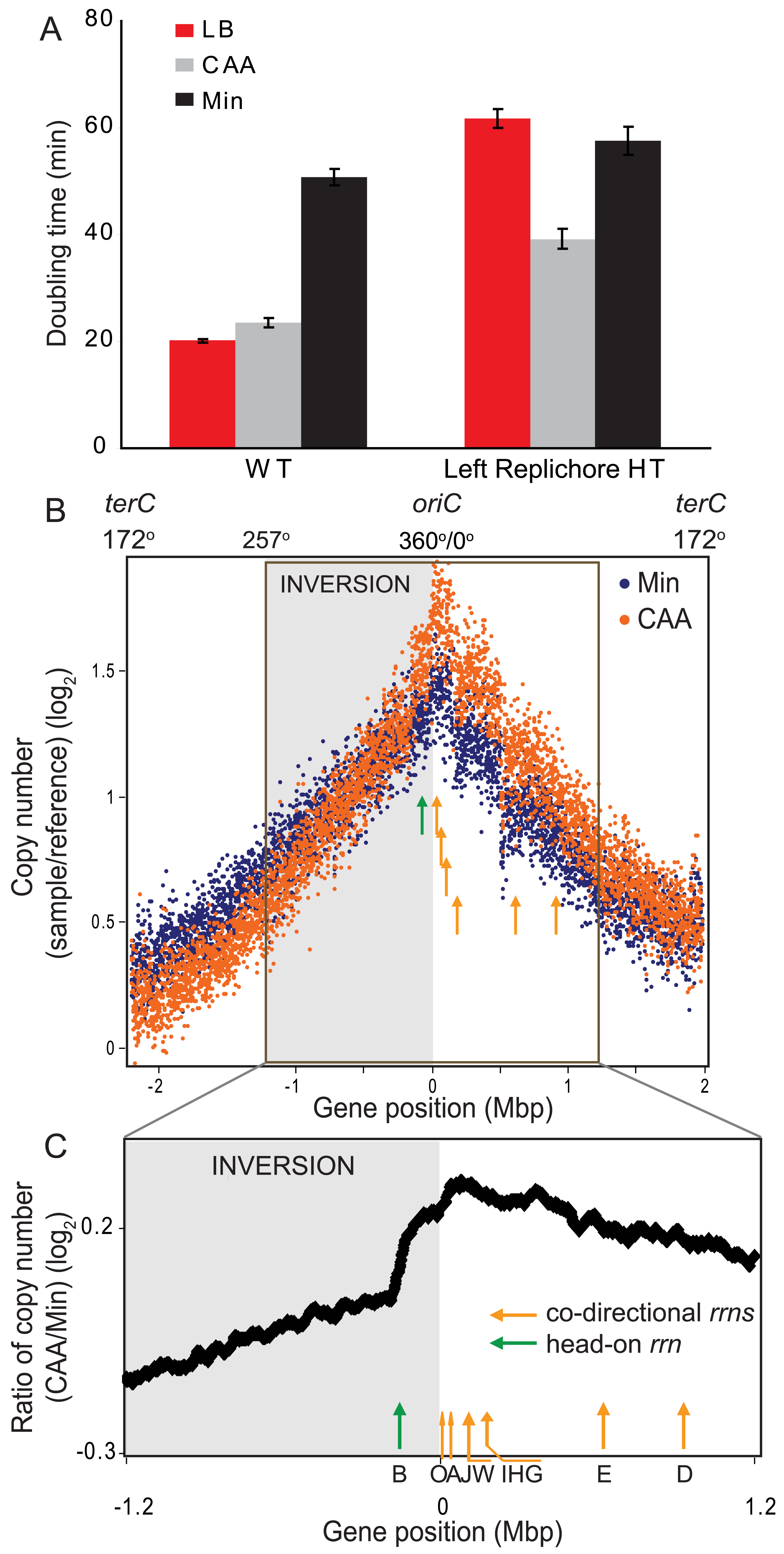

Supplement: Figure S1 — Inversion of the oriC-proximal half of the left replichore (HT-left) also leads to impedance of replication fork progression. (A) HT-Left exhibits a strong growth defect especially in rich media. Doubling times at 37°C in liquid LB (red bars), minimal medium with (CAA, grey bars), or without (Min, black bars) casamino acids were calculated by measuring OD600. (B) Overlay of the asynchronous genomic profiles of the HT strain grown in Min (blue), and CAA (orange). Profiles were obtained from asynchronous cultures grown at 37°C to OD600∼0.5. Average gene dosage ratios (log2) are plotted relative to gene positions adjusted according to known deletions of the background strain JH642 [6]. (C) Ratios of gene dosage (log2) in CAA versus Min of the HT-Left strain, calculated similarly to Figure 2C. Green arrow: position of the inverted rRNA operon rrnB; orange arrows: positions of co-directional rRNA operons; grey shaded region: inversion. (0.95 MB TIF) [file pgen.1000810.s001.tif]
