# Supplementary material for: Co-Orientation of Replication and Transcription Preserves Genome Integrity
Source: PLoS Genet. 2010 Jan 15;6(1):e1000810. doi: 10.1371/journal.pgen.1000810 (PMC2797598; doi:10.1371/journal.pgen.1000810)
Supplement: Table S1 — Comparison between the observed and expected fitness of the HT and UR mutants in the indicated growth media, under the multiplicative null model. (0.02 MB DOC) [file pgen.1000810.s002.doc]

**Table S1. Comparison between the observed and expected fitness of the HT and UR mutants in the indicated growth media, under the multiplicative null model**

| **Growth Medium** | **Observed Fitness ^a^**  **W_HT+UR_** | **Expected Multiplicative Fitness ^b^**  **W_HT_ W_UR_** | **P*-*value** |
| --- | --- | --- | --- |
| LB | 0.13440764 | 0.111940564 | 0.295089 |
| CAA | 0.20497558 | 0.237579801 | 0.323905 |
| Min | 0.563917744 | 0.656729304 | 0.011064 |

^a^: average fitness of the HT+UR mutant

^b^: average product of the observed relative fitness of the HT and UR strains
