# Supplementary material for: Co-Orientation of Replication and Transcription Preserves Genome Integrity
Source: PLoS Genet. 2010 Jan 15;6(1):e1000810. doi: 10.1371/journal.pgen.1000810 (PMC2797598; doi:10.1371/journal.pgen.1000810)
Supplement: Table S2 — Relative fitness of the HT strain in the indicated growth media (WHT). (0.03 MB DOC) [file pgen.1000810.s003.doc]

**Table S2. Relative fitness of the HT strain in the indicated growth media (W_HT_)**

| **Growth Medium** | **W_HT_ ^a^ (+/-SD)** | **W_marker_ ^a, b^ (+/-SD)** |
| --- | --- | --- |
| Min | 0.913 (+/-0.026) | 0.992 (+/-0.069) |
| LB | 0.190 (+/-0.063) | 0.938 (+/-0.084) |

**^a^**: relative to the wild type strain (JH642)

**^b^:** relative fitness of an *aprE::cat* strain measured to evaluate the effect of the *cat* marker

SD: standard deviation
