# Supplementary material for: Co-Orientation of Replication and Transcription Preserves Genome Integrity
Source: PLoS Genet. 2010 Jan 15;6(1):e1000810. doi: 10.1371/journal.pgen.1000810 (PMC2797598; doi:10.1371/journal.pgen.1000810)
Supplement: Table S3 — Strains. (0.05 MB DOC) [file pgen.1000810.s004.doc]

**Table S3**. Strains

| **Name** | **Description** | **Reference** |
| --- | --- | --- |
| JH642 | *trpC2 pheA1* | [1] |
| YB886 | *trpC2 metB5 sigB amyE spβ- ICEBs^o^ xin-* | [2] |
| JDW423 | JH642 *aprE::spc kan* inv(0°,94°) | This study |
| JDW424 | JH642 *aprE::oriC kan* Δ*dnaAN::spc* inv(0°,94°) | This study |
| JDW425 | JH642 *aprE::oriC kan* Δ*dnaAN::spc* | This study |
| JDW545 | JH642 *aprE::cat* | This study |
| JDW577 | JH642 *aprE::cat* inv(0°,94°) | This study |
| JDW605 | JH642 *argG::oriC kan* Δ*dnaAN::spc* inv (0°,257°) | This study |
| JDW712 | YB886 *aprE::cat* | This study |
| JDW713 | YB886 *aprE::cat* inv(0°,94°) | This study |
| JDW742 | YB886 *aprE::cat recA::*(*recA-gfp spc*) | This study |
| JDW743 | YB886 *aprE::cat* inv(0°,94°) *recA::*(*recA-gfp spc*) | This study |
| JDW858 | YB886 *kbaA'::neo' cat::'ybaN rrnG-5S'::erm 'neo::'ybaR* | This study |
| JDW859 | YB886 *rpsI'::neo' cat::'ybaJ rrnG-5S'::erm 'neo::'ybaR* | This study |
| JDW860 | YB886 *kbaA’::neo erm* inv(*ybaN::rrnG-5S*) *cat::’ybaR* | This study |
| JDW861 | YB886 *rpsI’::neo erm* inv(*ybaJN::rrnG-5S*) *cat::’ybaR* | This study |
| JDW878 | YB886 *kbaA'::neo' cat::'ybaN rrnG-5S'::erm 'neo::'ybaR recA::*(*recA-gfp spc*) | This study |
| JDW880 | YB886 *kbaA’::neo erm* inv(*ybaN::rrnG-5S*) *cat::’ybaR recA::*(*recA-gfp spc*) | This study |
| JDW946 | YB886 *aprE::cat tagC-gfp spc* | This study |
| JDW947 | YB886 *aprE::cat* inv(0°,94°) *tagC-gfp spc* | This study |
