# Supplementary material for: Co-Orientation of Replication and Transcription Preserves Genome Integrity
Source: PLoS Genet. 2010 Jan 15;6(1):e1000810. doi: 10.1371/journal.pgen.1000810 (PMC2797598; doi:10.1371/journal.pgen.1000810)
Supplement: Table S4 — Primers. (0.05 MB DOC) [file pgen.1000810.s005.doc]

**Table S4. Primers**

| **Name** | **Sequence** |
| --- | --- |
| oJW75 | agaagcaggtatggaggaac |
| oJW112 | tctcttgccagtcacgttac |
| oJW113 | atagatcgcctccatgaggt |
| oJW114 | cggcttctgaagccatgaac |
| oJW115 | gcagtggctgaatcttctcc |
| oJW135 | caaaactcggtttgctcaact |
| oJW146 | cggatcaaattgaaggtgag |
| oJW204 | gcgagtctagagtttattatgggccacgaaatgg |
| oJW205 | gagatccccgggagcccaagcgcatcattatatg |
| oJW206 | gcgagtctagatgctcacctcatttcttcacttc |
| oJW208 | gagatccccgggctcatattataaaagccagtcatt |
| oJW209 | aggtggaattcaactaaagcacccattagttca |
| oJW210 | aggtggaattccgtaccgtgagaactgccgt |
| oJW211 | aggtggaattcccgcatctgatgtctttgcttg |
| oJW360 | gagatccccgggttaatccgatacactgctgccgac |
| oJW388 | tgaaatcggcctccccaa |
| oJW428 | gtggtaagcttgagggctgtttctaacatt |
| oJW429 | gtggtgcatgcttactttgctgcaaact |
| oJW432 | gtggtggtaccttactttacctctgcggat |
| oJW433 | gtggtgagctccagaaccgatacttaatactctga |
| oJW434 | gtggtctgcagacgtcgtgagacagttcggtcccta |
| oJW435 | gtggtgtcgactttaaaaagcagaagcttgata |
| oJW436 | aggtgggatcctcaaaatggtatgcgttttgacaca |
| oJW437 | aggtggagctctgactagagaagaaagaatgaagattg |
| oJW438 | gtggtgagctccaatacaatccctagcatgatc |
| oJW439 | gtggtgaattcgaaaagaaaaacgaccgg |
| oJW442 | ccaattttcgtttgttgaac |
| oJW450 | agtctggatgagtctcccgtacaaactgc |
| oJW452 | gtgaatggaccaataataa |
| oJW485 | aggtgagatctatctgtgcggtatttcacacc |
| oJW486 | aggtgggtaccgtgtcggggctggcttaac |
