# Supplementary material for: Co-Orientation of Replication and Transcription Preserves Genome Integrity
Source: PLoS Genet. 2010 Jan 15;6(1):e1000810. doi: 10.1371/journal.pgen.1000810 (PMC2797598; doi:10.1371/journal.pgen.1000810)
Supplement: Text S1 — Supplemental Materials and Methods. (0.03 MB DOC) [file pgen.1000810.s006.doc]

**Supplemental Materials and** **Methods**

**Calculation of fitness and statistical analysis of epistasis**

To examine whether the effects of UR and HT on fitness are independent, we used a method for comparing effects of mutations at two different loci against the multiplicative model [3,4]. Namely, we compared W_HT+UR_ to W_HT_W_UR_, where W is the fitness of the UR, HT or HT+UR strain relative to the wild type strain. The relative fitness was calculated as the ratio of the Malthusian parameters, which are inversely proportional to the doubling times. The p*-*value was calculated using the Student’s t-test (paired two-sample, two tailed) based on replicated assays of fitness.

**Competition assay**

The inversion strain (resistant to chloramphenicol) was grown in coculture with the wild type strain. The competitors were preconditioned in the experimental medium by growing the mix to saturation density. This ensured that any difference in fitness was not due to a transition between their initial growth environments. Cultures were back-diluted 1:1000 everyday and allowed to grow to saturation, for approximately 32 generations. Samples were taken and plated on solid media with or without chloramphenicol, to determine the relative cell numbers of each competitor. Relative fitness was then calculated as the ratio of the number of doublings between initial and later samples for the two competing strains [5]. An *aprE::cat* strain was also grown in coculture with the wild type strain to evaluate the effect of the *cat* marker on fitness.

**References**

1. Dean DR, Hoch JA, Aronson AI (1977) Alteration of the Bacillus subtilis glutamine synthetase results in overproduction of the enzyme. J Bacteriol 131: 981-987.

2. Yasbin RE (1977) DNA repair in Bacillus subtilis. I. The presence of an inducible system. Mol Gen Genet 153: 211-218.

3. Segre D, Deluna A, Church GM, Kishony R (2005) Modular epistasis in yeast metabolism. Nat Genet 37: 77-83.

4. Elena SF, Lenski RE (1997) Test of synergistic interactions among deleterious mutations in bacteria. Nature 390: 395-398.

5. Lenski RE (1988) Experimental Studies of Pleiotropy and Epistasis in Escherichia coli. I. Variation in Competitive Fitness Among Mutants Resistant to Virus T4. Evolution 42: 425-432.

6. Srivatsan A, Han Y, Peng J, Tehranchi AK, Gibbs R, et al. (2008) High-precision, whole-genome sequencing of laboratory strains facilitates genetic studies. PLoS Genet 4: e1000139.
